# COVID‐19 and Pregnancy: Key Findings

**DOI:** 10.1111/sji.70109

**Published:** 2026-03-25

**Authors:** Gabrielle Gimenes Lima, Ana Flávia Segati, Giovanna Santos Oliveira, Nicoly Simões de Melo, Túlio Nakazato da Cunha, Elizabeth De Gaspari

**Affiliations:** ^1^ Immunology Center Adolfo Lutz Institute São Paulo Brazil; ^2^ InterUnit Post‐Graduation in Biotechnology University of São Paulo São Paulo Brazil

**Keywords:** immune response, immunisation, pregnancy, SARS‐CoV‐2, vaccine

## Abstract

Pregnant individuals were prioritised for COVID‐19 research due to concerns about increased susceptibility and limited clinical trial data. This narrative review synthesises evidence on maternal infection, immunological adaptations, placental susceptibility, and antibody transfer following maternal SARS‐CoV‐2 vaccination. Symptomatic COVID‐19 during pregnancy increases risks of severe outcomes, whereas vertical transmission remains rare. Placental pathology is characterised mainly by maternal vascular malperfusion and inflammation, with limited evidence of direct viral infection. Maternal vaccination—particularly with mRNA vaccines—induces robust IgG responses with efficient transplacental and lactational transfer, conferring passive neonatal protection. Key uncertainties include optimal vaccine timing, durability of neonatal immunity, and variant‐specific responses. Strengthening standardised research and ensuring inclusion of pregnant individuals is essential for global maternal health policy.

## Graphical abstract

Mechanistic overview of maternal SARS‐CoV‐2 immunisation, affinity‐matured IgG production, FcRn‐mediated transplacental IgG transport, and antibody delivery to the newborn via fetal circulation and breast milk. The schematic also highlights the translational relevance of murine models, which share conserved mechanisms of antibody induction despite structural differences in placental architecture.

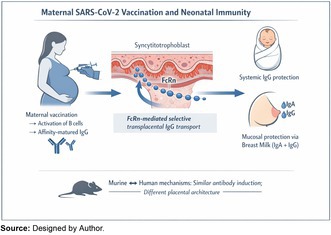

**Abbreviations**
ACE2: angiotensin‐converting enzyme 2
CDC: Centers for Disease Control and Prevention
CTSL: cathepsin L
DPP4: dipeptidyl peptidase‐4
EBCOG: European Board and College of Obstetrics and Gynaecology
FcRn: neonatal Fc receptor
IgA: immunoglobulin A
IgG: immunoglobulin G
IL‐6: interleukin‐6
mRNA: messenger
RCOG: Royal College of Obstetricians and Gynaecologists
RNANGF: Norwegian Society of Obstetrics and Gynaecology
SARS‐CoV‐2: severe acute respiratory syndrome coronavirus 2
TMPRSS2: transmembrane serine protease 2
WHO: World Health Organization

## 1 Introduction

Immunisation against SARS‐CoV‐2 has played a central role in global efforts to mitigate COVID‐19 morbidity and mortality [1]. From the onset of the pandemic, pregnant individuals were considered potentially vulnerable due to physiological and immunological adaptations that support maternal–fetal tolerance. Although early data were inconclusive, subsequent evidence demonstrated that symptomatic COVID‐19 during pregnancy increases the risk of severe disease compared to non‐pregnant individuals [2, 3, 4].

The pandemic also intensified psychosocial burdens for pregnant women, including stress, fatigue, and anxiety [5]. Meanwhile, rapid vaccine development raised questions about safety, immunogenicity, and the potential for passive immunity in newborns.

In humans, passive neonatal protection is primarily mediated by maternal–fetal IgG transfer via FcRn‐dependent placental transport [6], with transfer efficiency modulated by IgG subclasses and Fc‐glycosylation patterns [7]. Integrating experimental findings from murine models with observational human data strengthens mechanistic understanding of maternal–fetal immunity following SARS‐CoV‐2 vaccination.

Beyond prenatal IgG transfer, emerging evidence indicates that lactation also contributes to neonatal immunity, as breast milk contains virus‐specific secretory antibodies—predominantly IgA, but also IgG—that support mucosal protection in infants. Moreover, mRNA‐based vaccines demonstrated high effectiveness in the general adult population [8] and appeared safe in observational cohorts involving pregnant individuals [9, 10].

Preclinical animal studies have provided mechanistic insight into maternal immunisation. In our laboratory, female mice immunised with the SARS‐CoV‐2 receptor‐binding domain (RBD) antigen developed high titers of IgG antibodies and demonstrated transplacental transfer to offspring [11, 12]. Similar observations were independently reported using hACE2 mouse models [13, 14], supporting the use of murine systems to investigate placental antibody transfer.

## 2 Immunological Adaptations in Pregnancy Relevant to SARS‐CoV‐2

Pregnancy induces extensive immunological remodelling required to sustain a semi‐allogeneic fetus. Hormonal shifts—including progesterone and hCG—modulate immune cell function, promoting a predominantly anti‐inflammatory environment [15]. Regulatory T cells and NK cells undergo dynamic modifications that shape maternal susceptibility to infectious diseases [16].

Meta‐analytic evidence demonstrates increased risk of ICU admission, mechanical ventilation, and preterm birth in pregnant individuals with COVID‐19 [3, 17]. Severe maternal disease may induce cytokine dysregulation associated with adverse placental and neonatal outcomes [18].

## 3 Maternal Infection Outcomes and Placental Pathology

SARS‐CoV‐2 infects host cells via ACE2 and TMPRSS2. These factors show low co‐expression in the placenta, limiting susceptibility to direct viral infection [19, 20]. Alternative receptors, including DPP4 and CTSL, have been suggested [21, 22], though in vivo validation is limited.

Vertical transmission is rare, occurring in fewer than 1% of cases [23, 24, 25].

**FIGURE 1:**
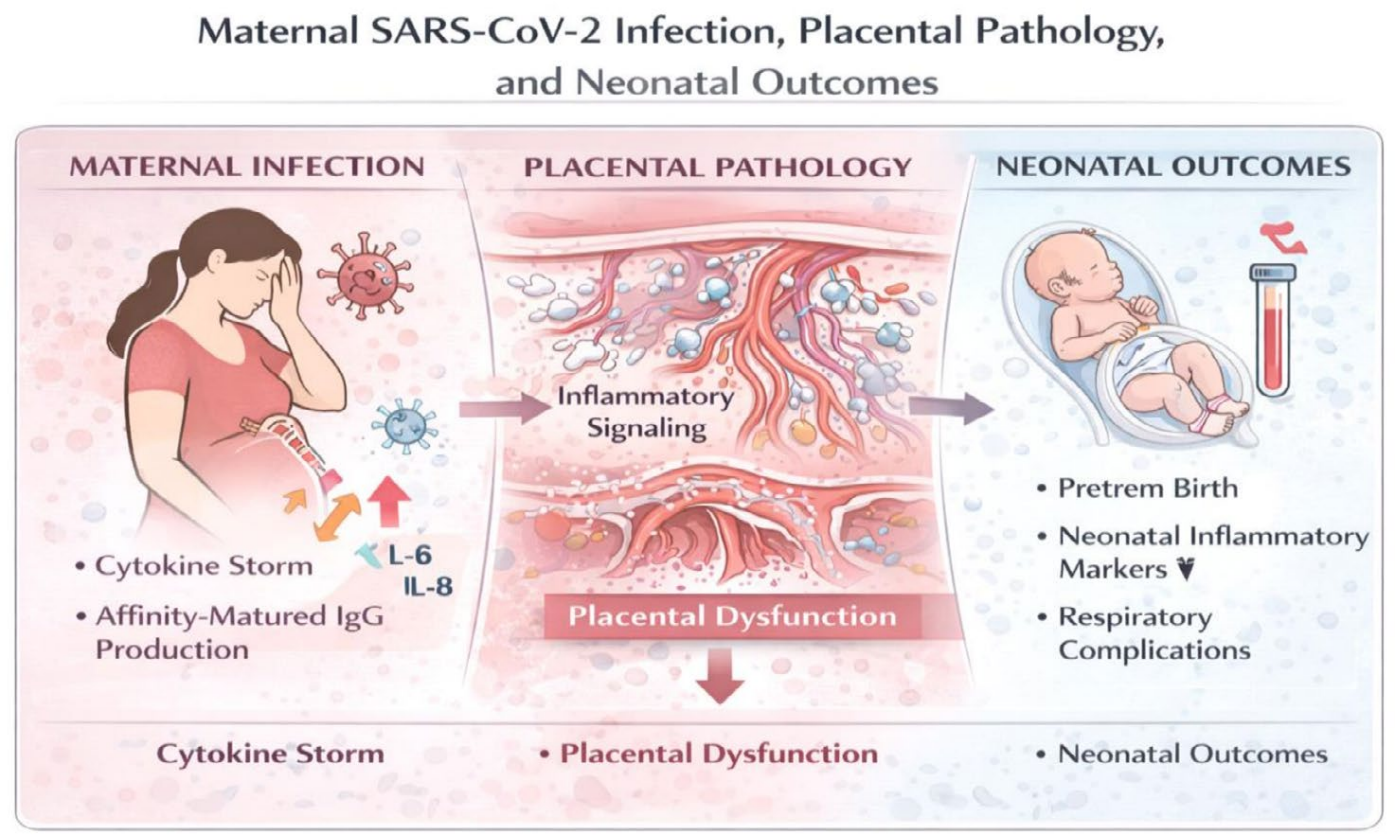
Maternal SARS‐CoV‐2 infection, placental pathology, and neonatal outcomes. Inflammatory cytokine surges during maternal infection contribute to placental dysfunction, leading to increased risks of preterm birth, neonatal inflammatory markers, and respiratory complications [30].

Placental pathology in SARS‐CoV‐2–affected pregnancies commonly includes maternal vascular malperfusion, fetal vascular malperfusion, and inflammatory infiltrates, as documented across several systematic reviews [26, 27, 28]. These changes often reflect systemic maternal inflammation rather than direct placental infection. The relationship between maternal infection, placental lesions and neonatal outcomes is summarised in Figure 1. These observations are consistent with broader reviews describing the clinical interplay between COVID‐19 and pregnancy outcomes [29].

## 4 Maternal Vaccination and Antibody Transfer (Placental and Lactational)

**FIGURE 2:**
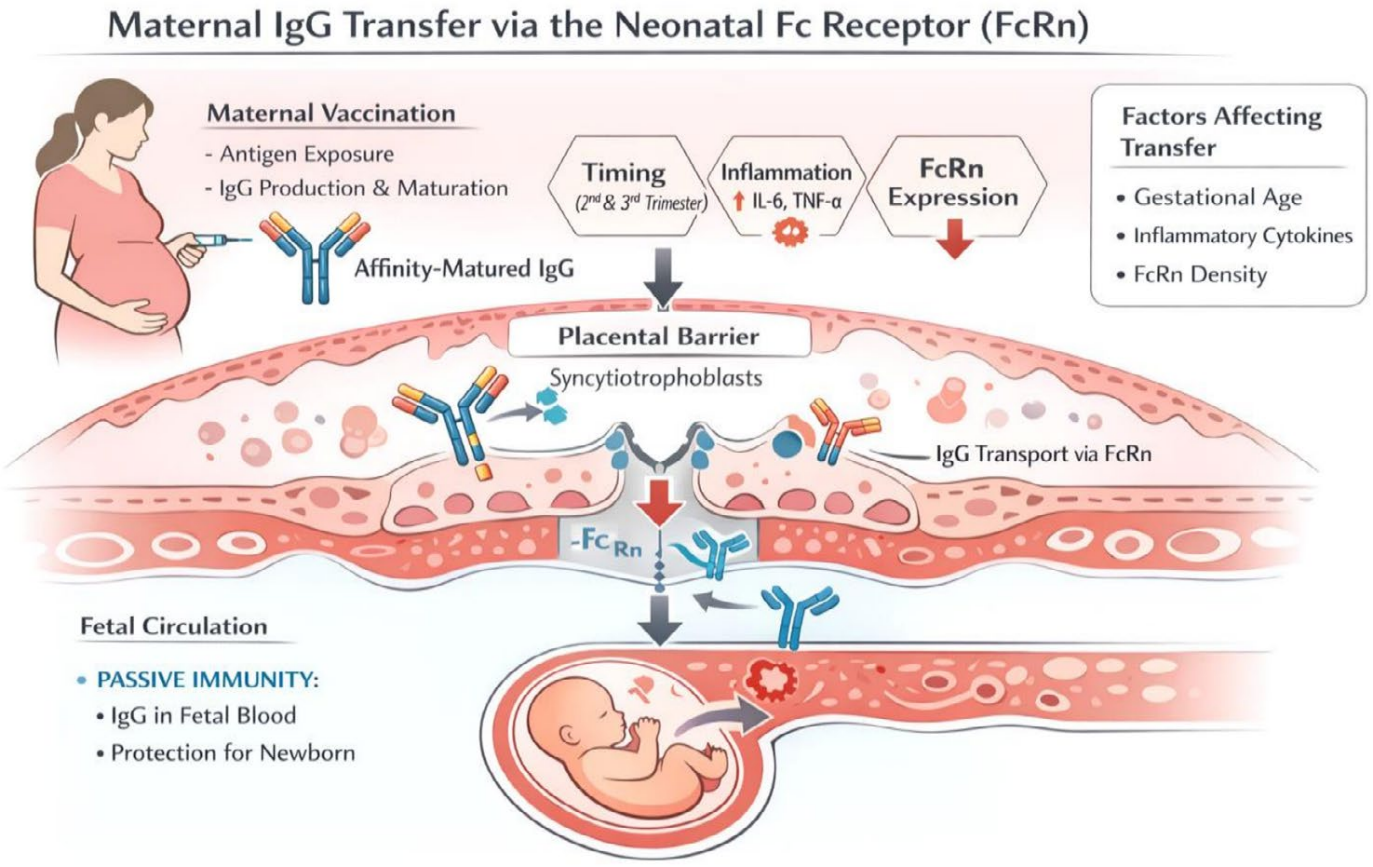
Maternal IgG transfer via the neonatal Fc receptor (FcRn). Maternal vaccination induces affinity‐matured IgG that binds FcRn at the syncytiotrophoblast surface, enabling selective transplacental transport into fetal circulation [6]. Transfer efficiency is modulated by gestational age, inflammatory cytokines (IL‐6, TNF‐α), and FcRn‐expression density. The fetus receives passive immunity through systemic IgG. Breast milk from vaccinated mothers contains SARS‐CoV‐2‐specific IgA and IgG, offering mucosal protection to infants [9].

Maternal vaccination reduces the risk of severe COVID‐19 during pregnancy and induces strong IgG responses that cross the placenta via FcRn‐mediated transport [9]. Cord blood studies demonstrate efficient transfer, especially when vaccination occurs in the second or third trimester. Effectiveness data from large population‐based studies reinforce the real‐world performance of COVID‐19 vaccines, including those administered during pregnancy [8]. Maternal vaccination induces strong affinity‐matured IgG responses that cross the placenta via FcRn (Figure 2). Breast milk contributes mucosal IgA and IgG, supporting dual‐layer neonatal immunity. Placental transfer efficiency is influenced by IgG subclasses and Fc‐glycosylation patterns, as demonstrated in recent human studies on Fc‐glycosylation and placental transfer [7].

Murine studies [11, 13] complement human observational data by demonstrating robust maternal antibody responses and maternal–fetal antibody transfer in a controlled experimental model, supporting mechanistic insights that are also observed in humans.

Mouse models are particularly informative in this context because they recapitulate key immunological events relevant to maternal immunisation, such as antigen‐specific B cell activation, affinity maturation of IgG, and FcRn‐mediated transfer to offspring, while acknowledging species‐specific differences in placental architecture and gestational timing.

Although placental architecture and gestational timing differ between species, these studies provide mechanistic insight into how maternal vaccination can prime neonatal protection through both prenatal and postnatal antibody exposure.

## 5 Integration of Animal and Human Data

### 5.1 Translational Considerations Between Animal Models and Human Pregnancy

**FIGURE 3:**
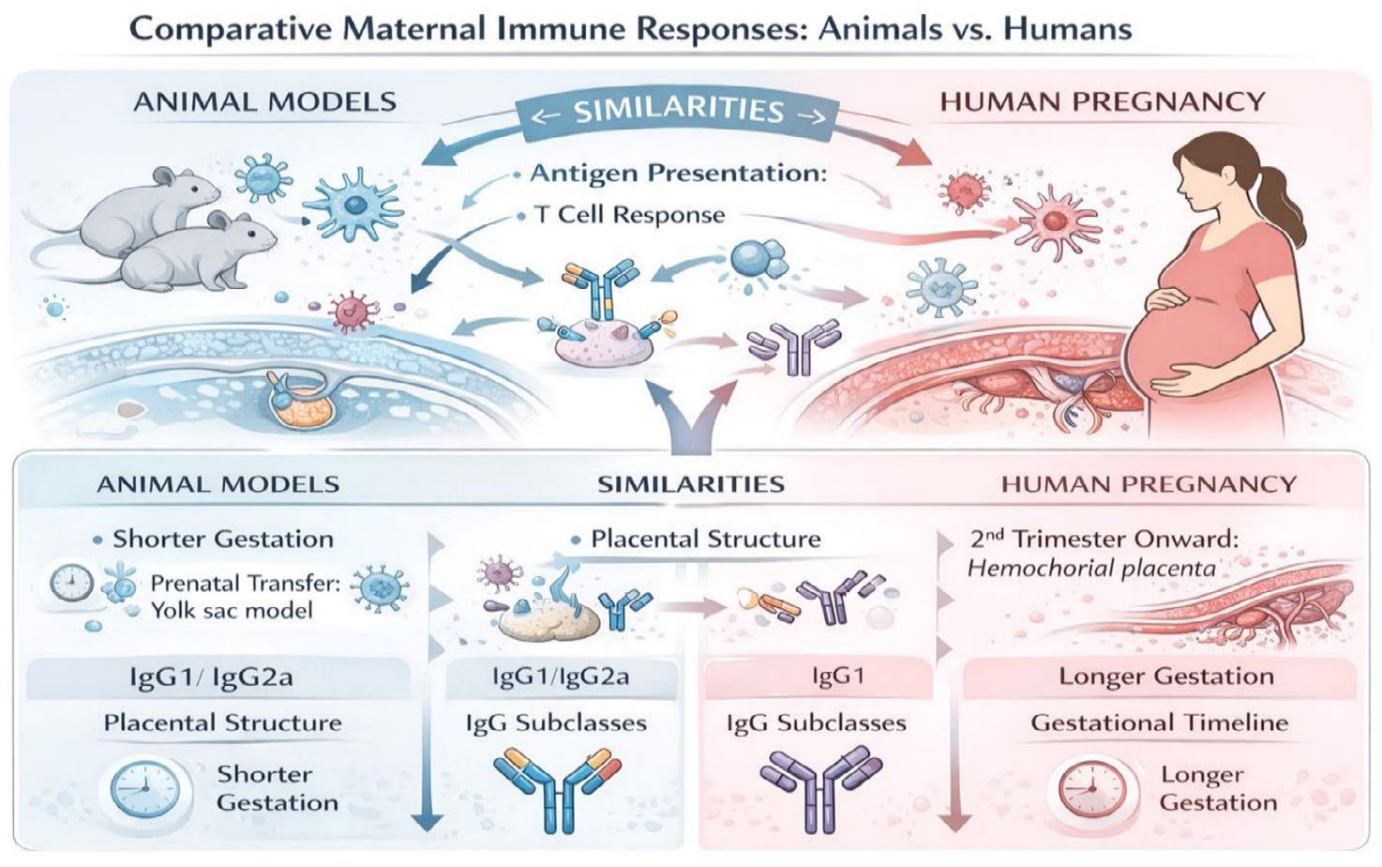
Comparative maternal immune responses in animal models vs. humans. Schematic contrast of immunological mechanisms in murine and human pregnancy, highlighting similarities in antigen presentation and IgG subclasses, while illustrating that in humans IgG1 is preferentially transported across the placenta, whereas other subclasses—particularly IgG2—show lower transfer efficiency [32]. Animal models display analogous FcRn‐dependent selectivity but with species‐specific subclass hierarchies. In humans, IgG subclass profiles and Fc‐glycosylation modulate placental transfer efficiency [7].

Preclinical studies in mice demonstrate robust maternal antibody production, placental transport, and neonatal immunity following SARS‐CoV‐2 vaccination [11, 13, 14, 31]. These mechanistic insights correspond to findings in human cohorts showing efficient FcRn‐mediated IgG transfer and protective antibody levels in cord blood and breast milk [9]. However, species‐specific differences—including placental architecture, gestational length, and timing of FcRn upregulation—necessitate cautious extrapolation. Animal models therefore serve as mechanistic complements to human observational studies rather than direct predictors of clinical outcomes. These similarities and differences between murine and human pregnancy are schematically summarised in Figure 3.

**FIGURE 4:**
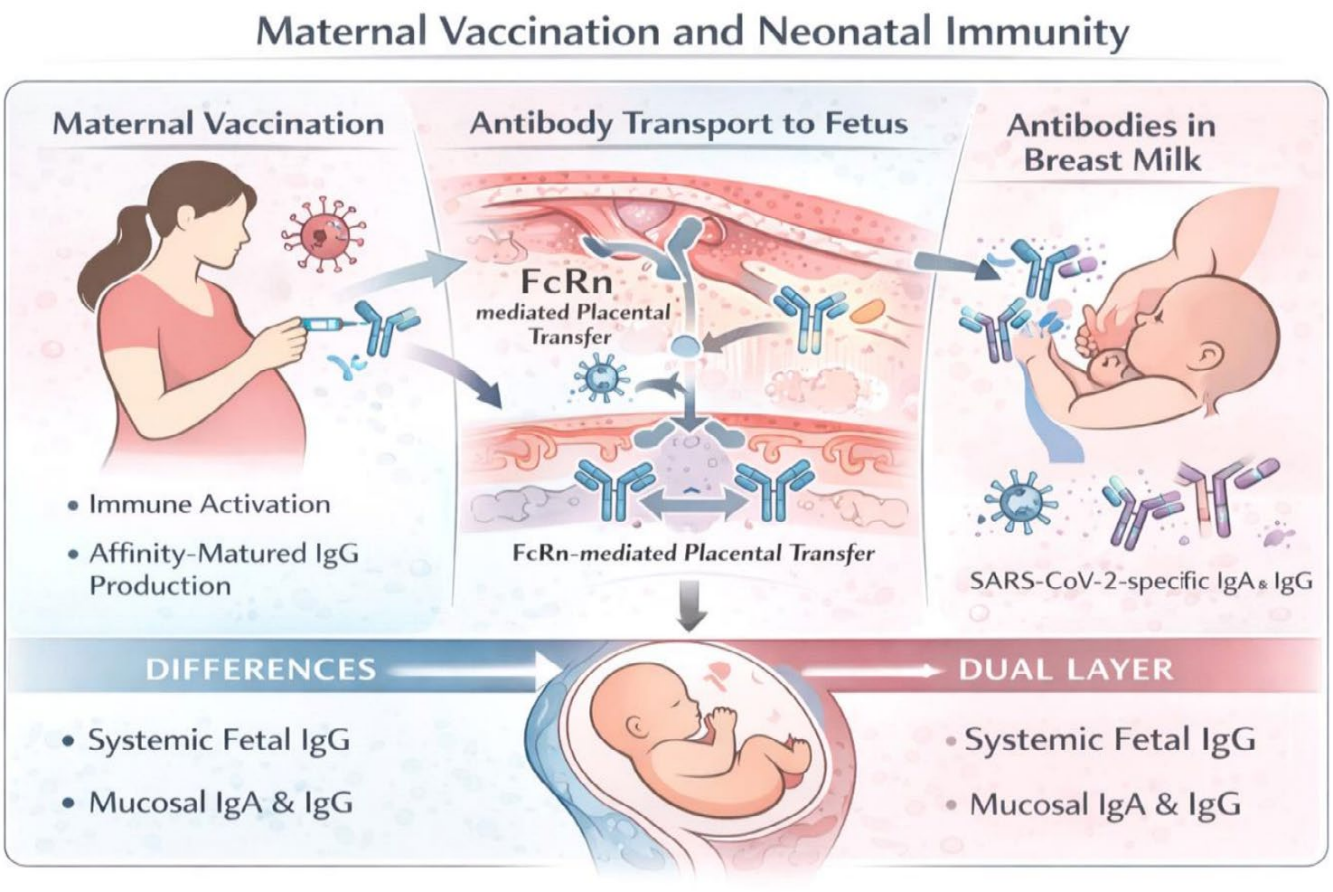
Maternal vaccination and neonatal immunity. Maternal vaccination triggers immune activation and affinity‐matured IgG generation. FcRn‐mediated transfer delivers IgG to fetal circulation, while breast milk provides SARS‐CoV‐2‐specific IgA and IgG. Newborn immunity consists of dual layers: systemic IgG and mucosal IgA/IgG. This mechanistic framework provides translational relevance, as murine models recapitulate key immunological events observed in human pregnancy, despite structural differences in placental architecture. Collectively, these findings support the translational bridge between mechanistic murine models and human observational studies.

An integrated overview of vaccine‐induced maternal and neonatal immunity in humans is depicted in Figure 4.

## 6 Summary of Human Vaccination Evidence

The main vaccines recommended for pregnant women are mRNA vaccines, such as BNT162b2, and viral vector vaccines. The most commonly used are the mRNA vaccines that work by providing genetic instructions that enable cells to produce a harmless viral protein, which stimulates an immune response without using a live virus. The relevance of using these vaccines during pregnancy lies in their ability to protect both the mother and the fetus from serious diseases, particularly infections such as COVID‐19, which can cause problems during pregnancy. Vaccination can also help transfer protective antibodies to the neonate, providing immunity early in life. Key findings from human studies evaluating COVID‐19 maternal vaccination, including vaccine platforms, gestational timing, antibody transfer, and safety outcomes, are summarised in Table 1 [8, 9, 10, 23, 33].

**TABLE 1:** Summary of key human studies evaluating COVID‐19 maternal vaccination.

Study	Vaccine platform	Gestational timing	Antibody transfer findings	Safety outcomes
Rosenberg‐Friedman et al. [9]	mRNA (BNT162b2)	Second–third trimester	High IgG and IgA levels in maternal serum, cord blood and breast milk	No maternal or neonatal adverse events
Lopez Bernal et al. [8]	mRNA; viral vector	Not pregnancy‐specific	High vaccine effectiveness supporting expected immunogenicity in pregnancy	No major safety concerns
Chen et al. [10]	mRNA; viral vector	Various	Efficient maternal–fetal IgG transfer; no evidence of vertical transmission	No increased pregnancy complications
Kotlyar et al. [23]	—	—	Vertical transmission rare (< 1%)	No consistent safety concerns
Anderson et al. [33]	mRNA	Third trimester	High neonatal IgG levels and protection against infection	No increase in adverse obstetric outcomes

## 7 Clinical and Policy Implications

International agencies—including WHO [34], EBCOG [35], RCOG [36], NGF [37], Brazil's Ministry of Health [38], and ANVISA [39]—support COVID‐19 vaccination during pregnancy. These recommendations are consistent with comparative analyses of immunisation strategies in pregnant women [40] and highlight:

- Reduction of severe maternal disease;
- Evidence of neonatal passive immunity; and
- Absence of significant safety concerns in large cohorts [10, 33, 40, 41].

Persistent vaccine hesitancy underscores the need for strengthened communication strategies and equitable access.

## 8 Conclusions and Future Directions

### 8.1 What Is Known

Maternal SARS‐CoV‐2 infection increases the risk of severe outcomes. The placenta shows low susceptibility to infection, and vertical transmission is rare. Maternal vaccination is safe, immunogenic, and confers neonatal protection through IgG transfer.

### 8.2 What Remains Uncertain

Optimal timing for vaccination to maximise neonatal immunity, durability of passive protection, and variant‐specific effects remains unresolved.

### 8.3 Research and Policy Priorities

Prospective standardised studies, harmonised placental evaluation protocols, and long‐term follow‐up of infants are needed. Global policies should aim to reduce disparities and support vaccine confidence.

## Author Contributions

G.G.L. and E.D.G.: conceptualisation. G.G.L. and E.D.G.: investigation. E.D.G.: resources. G.G.L. and E.D.G.: writing – original draft preparation. G.G.L, A.F.S., T.N.C., G.S.O., N.S.M., and E.D.G.: writing – review and editing. E.D.G.: supervision. E.D.G.: funding acquisition. All authors have read and approved the published version of the manuscript.

## Funding

This work was supported by Fundação de Amparo à Pesquisa do Estado de São Paulo (FAPESP) (grant number 18/04202‐0) and 21/11936‐3, Conselho Nacional de Desenvolvimento Científico e Tecnológico (CNPq) (grants number: 131308/2021‐1 and 305301/2022‐5), Coordenação de Aperfeiçoamento de Pessoal de Nível Superior (finance code 001) and CNPq to GSO 132059/2025‐8.

## Ethics Statement

The authors have nothing to report.

## Conflicts of Interest

The authors declare no conflicts of interest.

## Data Availability

Data sharing not applicable to this article as no datasets were generated or analysed during the current study.
